# Smaller meat portions contribute the most to reducing meat consumption in the United Kingdom

**DOI:** 10.1038/s43016-024-01070-2

**Published:** 2024-11-01

**Authors:** Alexander Vonderschmidt, Lindsay M. Jaacks, Peter Alexander, Rosemary Green, Alexandra L. Bellows, Cristina Stewart

**Affiliations:** 1 Global Academy of Agriculture and Food Systems, The University of Edinburgh, Midlothian, UK; 2 School of Geosciences, The University of Edinburgh, Midlothian, UK; 3 Centre on Climate Change and Planetary Health, Faculty of Epidemiology and Population Health, London School of Hygiene and Tropical Medicine, London, UK; 4 MRC/CSO Social and Public Health Sciences Unit, School of Health and Wellbeing, University of Glasgow, Glasgow, UK

**Keywords:** Risk factors, Climate-change mitigation, Psychology and behaviour, Agriculture, Human behaviour

## Abstract

Reducing meat consumption can help improve environmental and health outcomes, yet the effect of specific meat-reducing strategies is context dependent. Here, using decomposition analysis of National Diet and Nutrition Survey data (2008–2009 to 2018–2019), we found that in the United Kingdom, reduced meat portions had the largest impact on total meat consumption decline (52%), followed by fewer meat-eating days (24%), fewer meat consumers (17%) and fewer meat-eating meal occasions (7%). Understanding meat consumption behaviour patterns is key for more effective policies.

## Abstract (web summary)

Strategies to change meat consumption patterns remain poorly understood. Using decomposition analysis, this study shows that the decline in UK meat consumption is driven by the consumption of smaller portions of meat, followed by reducing the number of days and occasions on which meat is consumed.

## Main

High intake of meat, particularly red and processed meat, is associated with increased risk of many non-communicable diseases such as cardiovascular disease^1^, type 2 diabetes^2^ and some types of cancer (most notably, colorectal)^3^ and overall mortality^4^. Meat production is also the single largest contributor to greenhouse gas emissions, excessive land use and heightened freshwater withdrawals, all of which exacerbate climate change and deplete finite natural resources^5^. The UK Climate Change Committee (CCC) has therefore recommended a 20% reduction in meat consumption by 2030, rising to a 35% reduction by 2050 to reach net zero^6^. A recent analysis of meat consumption in the UK National Diet and Nutrition Survey (NDNS) revealed a gradual reduction between 2008–2009 and 2018–2019, from 103.7 g to 86.3 g per capita per day, or about 1.7% per annum^7^. This analysis found a reduction in red and processed meat consumption, together with an increase in white meat consumption, a trend which has also been observed by the Food Agriculture Organization^8^. In 2010, the Scientific Advisory Committee on Nutrition set the recommendation that adults in the United Kingdom with high intakes of red and processed meat (>90 g d^−1^) should consider reducing their intake to a maximum of 70 g d^−1^ (ref. ^9^). However, in 2018–2019, over one-third of adult meat consumers in the United Kingdom exceeded this recommendation^7^. Recent modelling work in Scotland—where meat intakes are comparable to the United Kingdom—suggests that further reducing red and processed meat intake to 60 g d^−1^ and 31 g d^−1^ would meet the CCC’s targets of a 20% and 35% reduction, respectively^10^.

Our study extends this earlier NDNS paper by examining the behaviours driving reductions in meat consumption, specifically quantifying changes in the proportion of the population who are meat consumers, the number of meat-eating days, daily meat-eating occasions and portion size of meat. By focusing on the specific habits and preferences that underlie meat consumption patterns, policymakers can more effectively design strategies that encourage sustainable dietary shifts and meet national reduction goals.

## Results

**Fig. 1:**
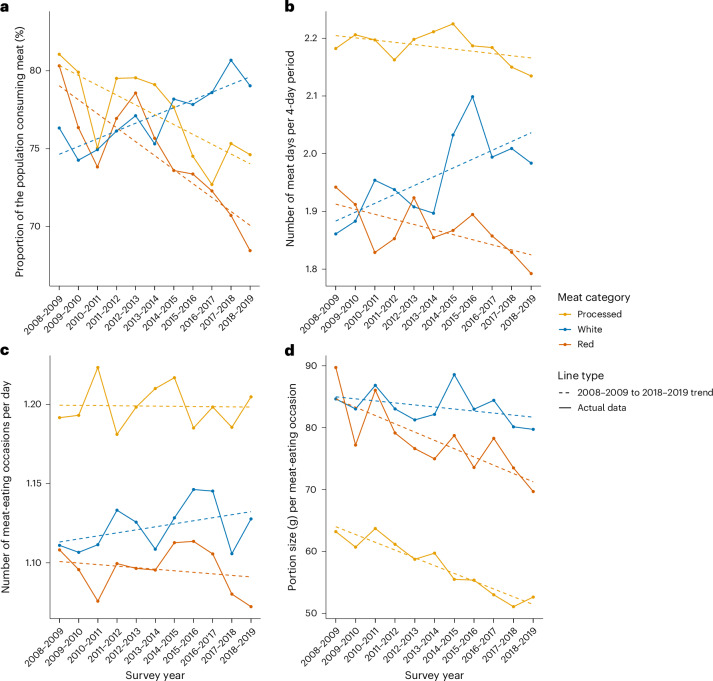
Trends of meat-eating behaviours in the UK NDNS rolling programme years 1–11. **a**, Proportion of the population consuming meat (%). **b**, Average number of meat-eating days over the four-day diary period. Meat-eating days (>0 g meat consumed) ranged from 0 to 4 days. **c**, Average number of meat-eating occasions per meat-eating day. Mean meat-eating meal occasions (containing >0 g meat) were within meat-eating days. **d**, Portion size (g) per meat-eating meal occasion. Mean portion size (g) of meat was across all meat-eating occasions. Trends over time were evaluated using Poisson regression models for count data (frequency of meat-eating days) and generalized linear regression models for continuous data (proportion of meat consumers, daily meat-eating occasions, portion size and per capita average consumption).

Our analytical sample consisted of 15,332 individuals in the NDNS aged 1.5–96 years who completed four food diary days (Supplementary Table 1). A full description of the data source, sample selection and analytical approach is detailed in Methods. In 2008–2009, 54% of the UK population consumed some type of meat on all four food diary days compared with 48% in 2018–2019 (Supplementary Fig. 1). From 2008–2009 to 2018–2019, the mean (standard error) daily per capita consumption of total meat decreased by 17.5 g from 103.4 (2.33) to 86.2 (2.65) (*P*_trend_ < 0.001) (Supplementary Table 2). The proportion of meat consumers dropped by 3.2% from 96.4 (0.74) to 93.4 (1.09) (*P*_trend_ < 0.001), whereas the average number of meat-eating days decreased by 0.15 days, from 3.39 (0.03) to 3.24 (0.04) (*P*_trend_ < 0.001), and the mean number of daily meat-eating occasions decreased by 0.03 occasions, from 1.49 (0.02) to 1.46 (0.02) (*P*_trend_ = 0.02). Mean portion size of meat decreased by 9.7 g, from 85.8 (1.73) to 76.1 (1.56) (*P*_trend_ < 0.001) in a meat-eating occasion (Fig. 1 and Supplementary Table 2). Similar trends were observed for red and processed meat, with the largest reductions observed for red meat (Fig. 1 and Supplementary Table 2). The only observed increases over time were for proportion of white meat consumers (+2.7%, from 76.3 (1.60) to 79.0 (1.65), *P*_trend_ < 0.001), white meat-eating days (+0.15 days, from 1.42 (0.04) to 1.57 (0.05), *P*_trend_ < 0.001) and daily white meat-eating occasions (+0.05 occasions, from 0.40 (0.01) to 0.45 (0.02), *P*_trend_ < 0.001). Daily per capita white meat consumption also increased (+3.15 g, from 32.59 (1.38) to 37.54 (1.65), *P*_trend_ = 0.001) (Supplementary Table 2). Mean portion size of white meat remained unchanged (84.7 g (2.52) to 79.7 g (2.41), *P*_trend_ = 0.18) (Fig. 1 and Supplementary Table 2).

Mean portion size of total meat decreased by 7.7 g at breakfast from 102.0 (6.02) to 94.3 (6.55) (*P*_trend_ = 0.008) and by 43.4 g at dinner from 284.9 (6.70) to 241.5 (7.34) (*P*_trend_ < 0.001), with no difference during lunch (*P*_trend_ = 0.13) (Supplementary Table 3).

There was a reduction in the proportion of meat consumers among all subgroups. Men further reduced their meat consumption through portion size reductions (−14.9 g, *P*_trend_ < 0.001), whereas women reduced their consumption across all remaining behaviours, with portion size decreasing at a slower rate than men (−4.48 g, *P*_trend_ = 0.02, *P*_int_ = 0.02) (Supplementary Table 4a). Adults further decreased their meat consumption across all remaining eating behaviours, whereas children only further decreased their meat consumption by portion size (−3.29 g, *P*_trend_ = 0.002) (Supplementary Table 4b). All three income tertiles further reduced their portion size (first: −11.7 g, *P*_trend_ = 0.01; second: −8.36 g, *P*_trend_ = 0.004; third: −7.42 g, *P*_trend_ = 0.006), with only the third (highest) income tertile also reducing their meat consumption through meat-eating days (−0.22 days, *P*_trend_ = 0.009) (Supplementary Table 5c).

**Fig. 2:**
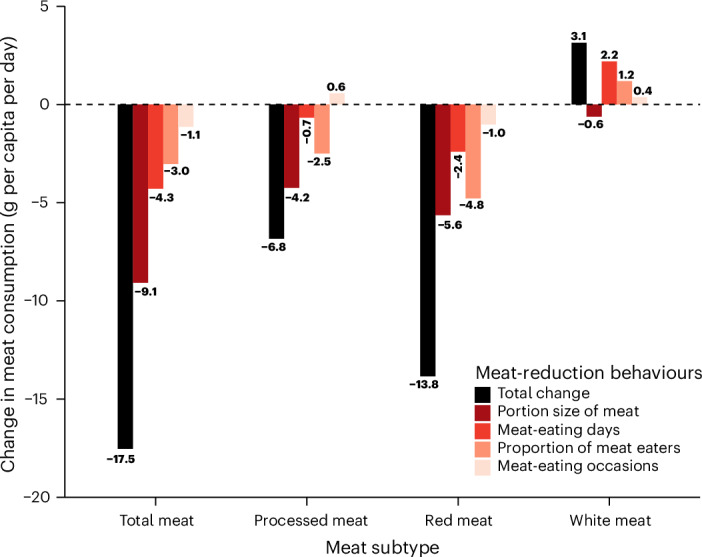
Decomposition analysis of meat-eating behaviours in the UK NDNS rolling programme years 1–11. Meat-eating days (>0 g meat consumed) ranged from 0 to 4 days. Mean meat-eating occasions (containing >0 g meat) were within meat-eating days. Mean portion size (g) of meat was across all meat-eating occasions. Note: each meat type is analysed as a separate population (for example, ‘processed meat consumers’, ‘red meat consumers’ and so on).

In decomposition analysis, of the 17.5 g decrease in per capita total meat consumption, portion size contributed 51.8% (−9.07 g), the number of meat-eating days contributed 24.4% (−4.28 g), proportion of meat consumers contributed 17.3% (−3.03 g) and the number of daily meat-eating occasions contributed 6.5% (−1.14 g) (Fig. 2 and Supplementary Table 5). In subgroup analyses, portion size remained the largest contributor among all groups (men: 69.4%, women: 38.5%, adults: 48.1%, children: 66.3%, first income tertile: 54.8%, second income tertile: 73.8%, third income tertile: 45.4%) (Supplementary Table 5).

## Discussion

This study investigates changes in meat consumption behaviours over time in the UK population, offering insights not previously explored. Reducing portion sizes of meat, particularly for red and processed meat, was the largest driver of recent meat consumption declines between 2008–2009 and 2018–2019.

Our finding that reduced portion sizes were the primary factor in declining meat consumption aligns with previous research suggesting smaller portion sizes may be an effective strategy to reduce meat intake^11^. Indeed, previous research has highlighted that meat-orientated consumers (compared with flexitarians) have the lowest appreciation of meat-free meals, and as such, encouraging this group to reduce their portion sizes of meat may be most impactful^12^. Transitioning to low-meat meals might also present fewer barriers than adopting meat-free meals due to reduced reliance on taste preferences, perceived social norms and cooking skills, for example^13^. More broadly, these findings are corroborated by previous literature identifying mechanisms underlying the effectiveness of portion size reductions in lowering energy intake. For example, segmenting meat portions into smaller units has been found to subtly reduce energy intake without overwhelming consumers, leveraging ‘unit bias’—where individuals may consume less when food is presented in smaller units^14^. This observation aligns with existing research, which also identifies portion size reductions as the primary driver for decreased meat consumption among groups traditionally identified as high consumers and more resistant to reducing meat intake, notably men^15^ and lower socioeconomic status groups^16^. Conversely, women and those in higher income groups—who are more likely to be low consumers and more open to vegetarianism^16^—reduced their meat intake across all behaviours including meat-free days and meat-free meal occasions, representing a more deliberate effort. An earlier analysis of NDNS data found that both men and women decreased their meat consumption at a similar rate^7^, emphasizing that simply examining overall changes in meat consumption overlooked these meaningful behavioural differences related to gender. Whereas our findings are based on observed consumption behaviours and not experimental trials, they support the possibility of interventions and policies aimed at reducing meat portion sizes to accelerate meat-reduction efforts. This evidence also suggests that interventions designed to specifically target high consumers (that is, men and lower socioeconomic status groups) would be appropriate. Collaboration between policymakers and the food industry to establish guidelines for meat portion size, reformulate composite meat dishes and develop both hybrid meat products and blends of meat with high-protein plant-based ingredients could be an important step forward.

We observed a reduction in total meat portion size by 9.7 g over the decade, approximately 2% per annum. Considering established links between standard serving sizes for red (100 g, 3.5 oz) and processed (50 g, 1.8 oz) meat and non-communicable diseases^1–4,17^, these reductions appear modest. Dose-response relationships between red and processed meat consumption and non-communicable diseases are often described as serving-size increments in previous studies^2,4,17^. Notably, red and processed meat consumption in the NDNS averaged below one serving daily. Therefore, analyses exploring associations between meat consumption and non-communicable disease might benefit from more detailed dose-response curves featuring increments smaller than standard serving sizes. In contrast to reductions for red and processed meat across all consumption behaviours, white-meat-eating days and occasions and per capita white meat consumption increased. This divergence may be attributed to several factors. First, the trend of increased white meat consumption could be driven by decreasing costs relative to other meats. For example, in the United Kingdom, roasted chicken prices fell from 304 pence kg^−1^ in December 2008 to 278 pence kg^−1^ in December 2018, whereas over that same period, beef mince prices rose from 595 pence kg^−1^ to 671 pence kg^−1^ (ref. ^18^). Second, white meat may be perceived as a healthier alternative considering that unlike red and processed meat, it has not been classified as a carcinogen by the World Health Organization’s International Agency for Research on Cancer^17^. Moreover, white meat consumption has shown mixed associations on health outcomes such as cardiovascular disease^19^, lacking the clear causal links observed with processed meat^20^. Last, environmental concerns associated with meat consumption predominantly target beef due to its notably higher greenhouse gas emissions—nearly ten times higher compared with poultry^5^. Consequently, individuals motivated by environmental concerns may be opting for white meat as a less impactful alternative.

In light of portion size reductions primarily driving declines in meat consumption among individuals in the United Kingdom, emphasis could shift to promoting complementary approaches, such as meat-free meals and meat-free days. We found that meat-free days notably drove reduced meat consumption among women and those in the highest income group. Similarly, studies of participation in ‘Meatless Monday’ in New York and France have shown that participants were more likely to be women and higher income^21,22^, suggesting that certain groups may respond more positively to these higher-friction reduction efforts. Furthermore, Meatless Monday campaigns have gained widespread adoption in over 40 countries, garnering support from various public health and environmental organizations^23^. Leveraging such campaigns could effectively raise awareness and bolster participation in meat-reduction efforts^24^.

A key strength of our study is the use of the NDNS, the only dataset capturing nationally representative diet data for the United Kingdom. Additionally, introducing a mathematical decomposition technique highlighted the relative importance of different dietary behaviours. A limitation of our study is the cross-sectional nature of the NDNS, which precludes the examination of individual longitudinal dietary behaviour changes. Furthermore, this study utilized NDNS survey data up to 2019; data for 2020 are also available but were collected during the COVID-19 pandemic, which were not representative and used a modified dietary assessment method^25^. It is also important to note that in the absence of self-reported meal occasions in the NDNS, we defined meal occasions using a previously published approach—on the basis of a minimum time interval^26^. This approach may not accurately reflect participants’ true meal occasions; different definitions of a ‘meal’ could be applied, potentially yielding different results. Misreporting and underreporting are also inherent limitations of self-reported dietary assessment methods^27^. The NDNS attempts to address this by providing participants with guidance, including photographic examples of commonly consumed foods with labelled portion size variations for increased accuracy when estimating portion size of foods consumed^28^. Specific to the subgroup analysis involving children, dietary assessment is complicated by the requirement for parents or carers to complete the four-day food diary for those aged ≤11 years, with input from the child where appropriate. However, any potential bias that would be introduced by this method of data collection for children is assumed to be consistent across the survey years, which may not considerably distort longitudinal trends.

In conclusion, nationally representative dietary data suggest that declining meat consumption trends in the United Kingdom are being predominantly driven by reductions in meat portion sizes, with significant variations across gender, age and income groups. Future research may wish to explore underlying mechanisms of the reductions in portion size (or other consumption behaviours) through specific food types (for example, composite dishes vs individual meat items). Additionally, assessing how these reductions align with overall caloric intake and dietary trends could provide deeper insights into the broader nutritional implications of these changes. It’s also important to determine where these meals are consumed—whether at home or out of home—to understand whether portion size reductions stem from broader population-level changes such as restaurant or retailer adjustments, or individual choices. These findings emphasize the potential of targeted portion size interventions and public health policies as an effective strategy for accelerating reductions in meat consumption towards health and environmental goals.

## Methods

This study did not require ethical or regulatory approval as it utilized publicly available, anonymized data from the UK National Data Service. The NDNS rolling programme adheres to the Declaration of Helsinki and operates under the UK’s Health Research Authority Research Ethics Committee; approval references: #07/H0604/113 (Years 1–5) and 13/EE/0016 (Years 6–11)^29^. Data collection for the NDNS was conducted in compliance with ethical regulations, as described in the NDNS documentation^29,30^. Participants received up to £50 compensation for their time and participation in the survey: £20 for providing a blood sample during the nurse visit and £30 for completing at least three of four food diary days^30^. Informed consent was obtained from all participants—or their guardian(s), as appropriate—as part of the original NDNS data collection process^29^.

### Data source and sample

The NDNS rolling programme is a continuous, cross-sectional survey, which collects detailed dietary intake data and nutritional status information from the UK population aged 1.5 years and older, living in private households^28–30^. The NDNS is funded by Public Health England and the UK Food Standards Agency. It aims to monitor the diet and nutrition of the UK population, providing evidence on adherence towards public health nutrition targets, ensuring ongoing governmental support and resources for the collection and analysis of nutritional data. Further information on the NDNS methodology, including survey design and weighting, have been previously described^30^. Briefly, the NDNS is designed to be nationally representative of the UK population and adjusts for age and sex population distributions through survey weighting. The sample was drawn from Postcode Address Files, which were grouped into Primary Sampling Units (PSUs) based on postcode sectors. From each PSU, a list of addresses was randomly selected, and the interviewer randomly selected up to one adult and one child to take part from each household. This study included survey participants from Years 1–11 (2008–2009 through 2018–2019) of the rolling programme.

### Dietary intake data

Dietary data were collected using four-day, consecutive food diaries, with the survey design ensuring equal representation of all days of the week. Detailed methodology on NDNS data collection has been described elsewhere^28^. Briefly, participants were instructed to record all food and beverages they consumed over the assigned four-day period within a paper journal. For children aged ≤11 years, a parent or carer was asked to complete the four-day food diary with input from the child as appropriate. Children ≥12 years were asked to complete the diary themselves, with details confirmed with others where necessary^28^. Participants estimated portion sizes using household measures (for example, tablespoons) or reporting the weights on food labels. As this study explored the frequency of days in which meat was consumed, participants with <four food diary days were not included in the analyses (*n* = 323, 2%).

### Meat categorization

We explored the consumption of total meat and processed meat, red meat and white meat separately. We did not include fish consumption in this analysis. Estimates of meat intake were based on disaggregated data where all non-meat components of composite dishes were excluded (that is, the grams of beef only were estimated in beef lasagne)^28,31^. Meat items were disaggregated into pre-existing categories within the NDNS. For dishes containing more than one type of meat, each meat type was disaggregated separately, into one of 11 mutually exclusive categories. We grouped these categories into processed, red and white meat, aligning with the approach used in a previous trend analysis of meat consumption in the NDNS^7^:

1. Processed meat—processed red meat, processed poultry, sausages and burgers.
2. Red meat—beef, lamb, pork, other red meat and offal.
3. White meat—poultry and game birds including duck.

### Meat consumption behaviours

We assessed how meat intake changed over time, and specifically explored the change in four distinct meat consumption behaviours: (1) proportion of the population consuming meat, (2) frequency of meat-eating days for meat eaters; (3) daily meat-eating occasions for days where meat is eaten and (4) portion size (in grams) of meat within meat-eating occasions. For dishes containing more than one meat subtype (that is, processed, red and white meat), frequency of consumption and portion size of each subtype was established separately. For example, ‘chicken, bacon and mushroom cream pie’, was considered both a white meat and processed meat item. Consequently, the portion sizes of both white meat and processed meat were estimated separately and the frequency of consumption for each subtype was counted independently.

The proportion of the population consuming meat was calculated through a survey-weighted ratio of meat consumers (>0 g) to non-consumers (0 g).

Meat-eating days were defined as the number of days in which any quantity of meat (>0 g) was consumed across the four-day food diary period among meat consumers. We also explored the number of days in which no meat was consumed and investigated the distribution of individuals who ate meat on 0, 1, 2, 3 and 4 days across the four-day period.

Eating occasions were defined as intake ≥50 kcal (from all food and drink) recorded with an interval of >30 minutes between eating^26^. We defined a meat-eating occasion as intake ≥50 kcal (from all food and drink) and >0 g of meat recorded with an interval of >30 minutes between eating. Average daily meat-eating occasions was calculated as participants’ mean daily number of meat-eating occasions, across all meat-eating food diary days.

We determined the portion size of meat (in grams) consumed during each meat-eating occasion. The mean meat portion size was calculated by averaging the grams of meat consumed across all meat-eating occasions. Further, we investigated portion size of meat consumed by standard mealtime: breakfast (6:00–11:00 a.m.), lunch (12:00–3:00 p.m.) and dinner (4:00–11:00 p.m.), mirroring time periods of a previous mealtime analysis in the NDNS^26^.

### Sociodemographic characteristics

Sociodemographic variables included self-reported age, sex and equivalized household income tertiles. For age, participants were asked to provide their date of birth, or age at last birthday if unknown; with interviewer estimates used if participants were unable or unwilling to provide this information. We categorized participants into the age groups of children (<18 years) and adults (≥18 years). Participants were asked to self-identify as either male or female, and in cases of non-disclosure, the interviewer reported sex. Participants reported their total household income from the previous 12 months, before deductions and tax, inclusive of housing benefits and child allowance. Within the NDNS data files, these data were equivalized, accounting for household size and composition and split into tertiles^32^.

### Statistical analysis

To address clustering within the sample and minimize potential selection and non-response bias at both the household and individual level, our analyses included survey weights and PSUs published in the NDNS datasets and additional clustering at the household level.

We report the proportion of meat consumers, mean number of meat-eating days, mean number of daily meat-eating occasions per capita and the mean portion size of meat within a meat-eating occasion in each survey year. We also report the daily per capita average of meat consumption. We investigated trends over time (2008–2009 to 2018–2019) using Poisson regression models in analyses of count data (frequency of meat-eating days) and generalized linear regression models for continuous data (proportion of meat consumers, daily meat-eating occasions, portion size and per capita average consumption). Additionally, we conducted separate univariate analyses for each population subgroup, considering factors such as sex, age group and equivalized household income tertiles. In these models, confidence intervals for the coefficients were calculated using the profile likelihood method, implemented by the confint() function in base R and exponentiated for interpretability where applicable.

To estimate the proportion of responsibility for each meat consumption behaviour relative to the overall decrease in consumption^7^, we used a decomposition analysis, based on the following equation implemented by Alexander et al.^33^.

$$\Delta X=\left(\frac{{c}_{i,t_2}-\,{c}_{i,t_1}}{\mathrm{ln}\left({c}_{i,t_2}\right)-\mathrm{ln}\left({c}_{i,t_1}\right)\,}\right)\mathrm{ln}\left({X}_{i,t_2}/{X}_{i,t_1}\right)$$

Here *c*_*i*,*t*_ represents the average total meat consumed per capita *i*, at time *t* (where *t*_1_ is baseline and *t*_2_ is the subsequent survey year), and *X* represents (separately): the proportion of meat consumers, the mean number of meat-eating days, the mean number of daily meat-eating occasions and the average portion size of meat (g) within a meat-eating occasion. We also ran the decomposition analysis by sex, age group and equivalized income tertile.

All analyses were performed in R version 4.2, using the ‘survey’ and ‘srvyr’ packages to account for survey weighting in the demographic and regression analyses. *P* < 0.05 was the criterion for statistical significance in trend analyses and *P* < 0.1 for the subgroup interactions.

### Reporting summary

Further information on research design is available in the Nature Portfolio Reporting Summary linked to this article.

## Supplementary information

Supplementary Information Supplementary Figs. 1 and 2.

Reporting Summary

Supplementary Tables Supplementary Tables 1–5.

## Data Availability

This analysis used data from the National Diet and Nutrition Survey (NDNS) rolling programme years 1–11 (2008–2009 through 2018–2019). These data are open access and freely available for download from the UK Data Service: https://beta.ukdataservice.ac.uk/datacatalogue/series/series?id=2000033. NDNS data are recommended to be downloaded directly from the UK Data Service so that any pertinent updates and data sharing agreements are directly available for the downloader/user.

## Code availability

All code used for analysis in this paper is open access via GitHub at https://github.com/axvonder/NDNSMeatTrends.
