# Supplementary material for: Smaller meat portions contribute the most to reducing meat consumption in the United Kingdom
Source: Nat Food. 2024 Nov 1;5(12):982–7. doi: 10.1038/s43016-024-01070-2 (PMC11655353; doi:10.1038/s43016-024-01070-2)
Supplement: Supplementary file 1 — Supplementary Figs. 1 and 2. [file 43016_2024_1070_MOESM1_ESM.pdf]

nature food

Supplementary information

https://doi.org/10.1038/s43016-024-01070-2

Smaller meat portions contribute the most to reducing meat consumption in the United Kingdom

In the format provided by the authors and unedited

Supplemental Figures for *Smaller meat portions contribute the most to reducing meat consumption in the UK*

SI Figure 1. Proportion of respondents by number of meat-eating days over time in the UK National Diet and Nutrition Survey rolling programme years 1-11 (2008/09 to 2018/19).

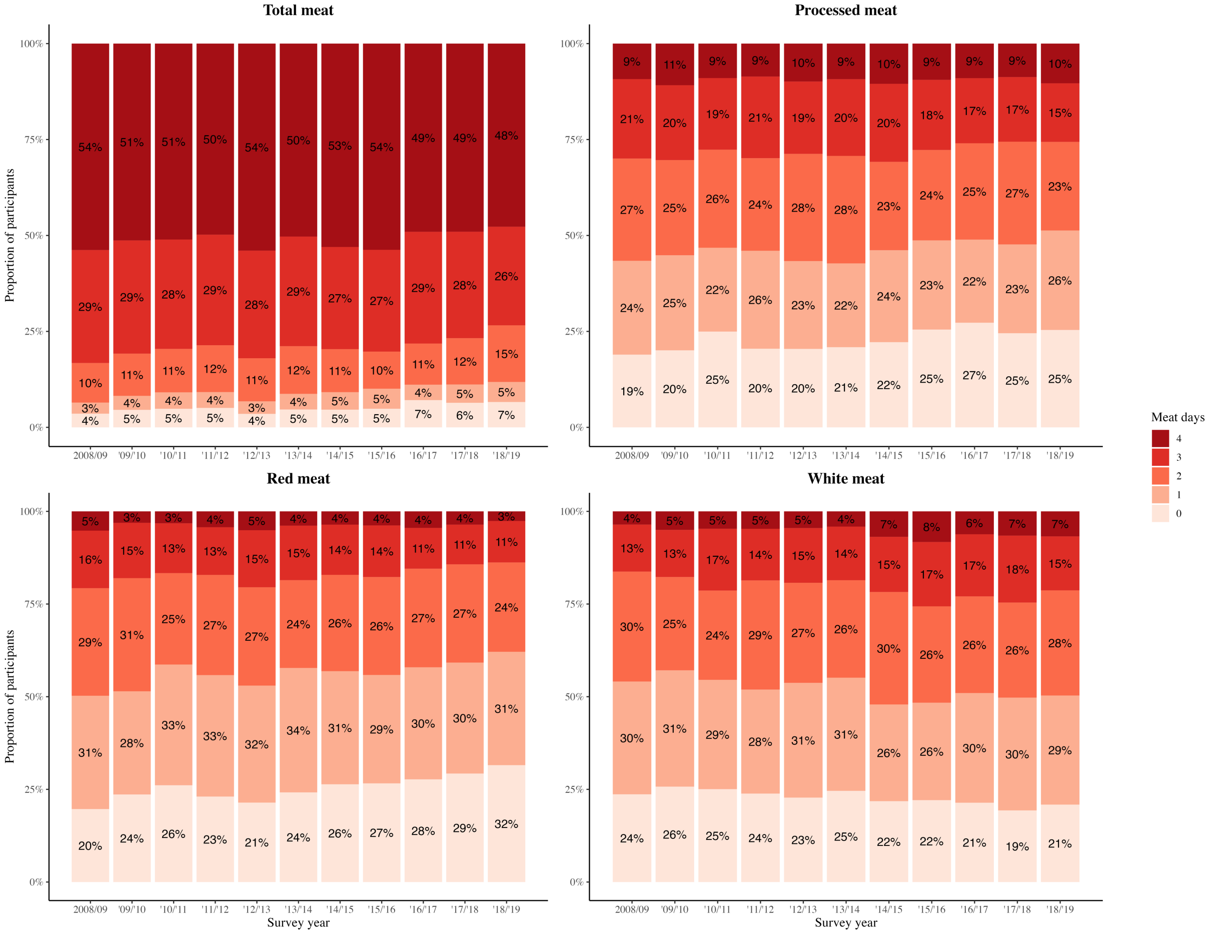

Meat-eating days ranged from 0 to 4 days. Meat-eating days were defined as the number of days in which any quantity of meat (>0g) was consumed across the 4-day food diary period.

SI Figure 2. Trends of meat-eating days, meat-eating occasions, and meat portion size for total meat only in the UK National Diet and Nutrition Survey rolling programme years 1-11 (2008/09 to 2018/19).

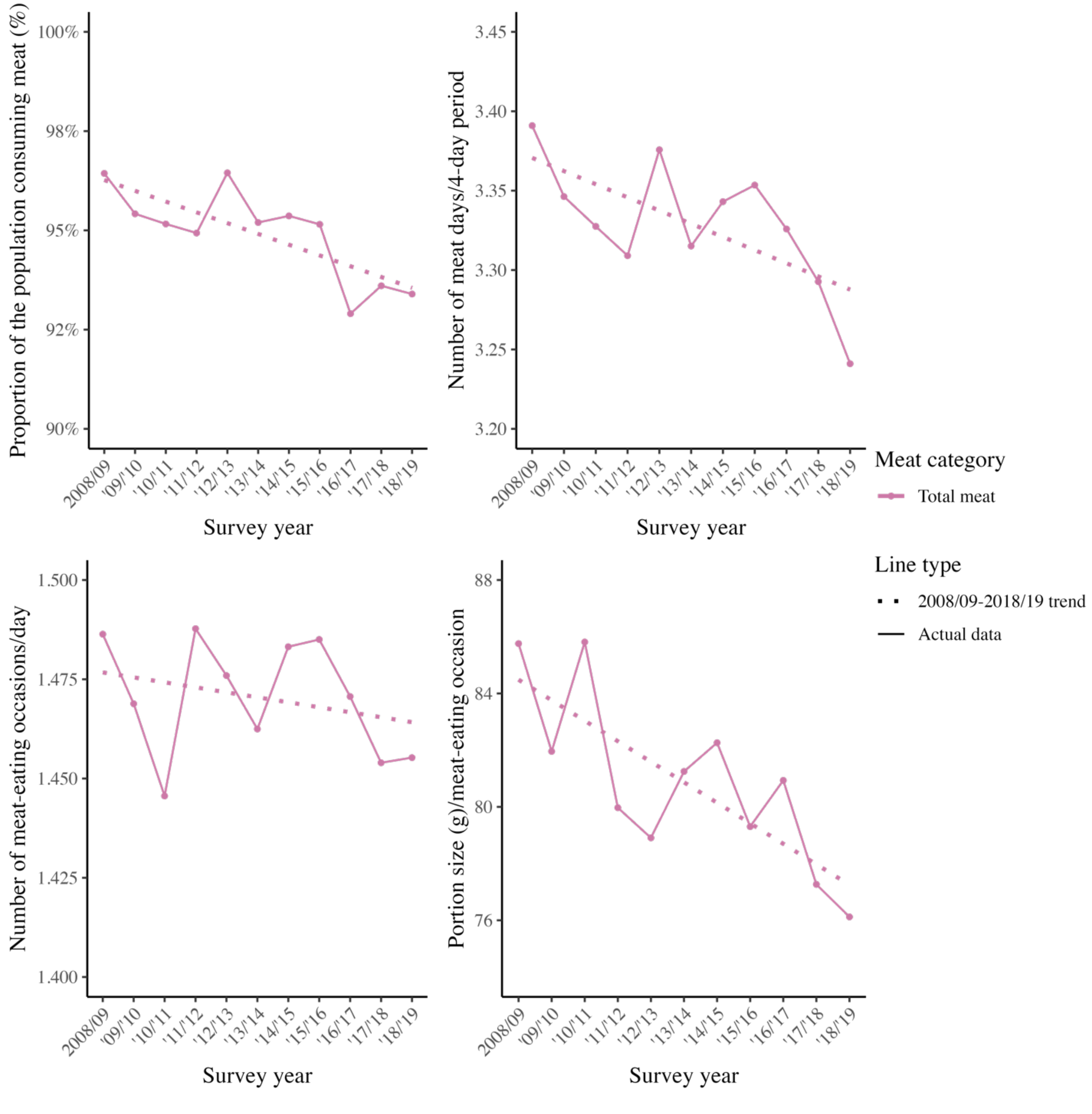

Meat-eating days (>0g meat consumed) ranged from 0-4 days. Mean meat-eating occasions (containing >0g meat) were within meat-eating days. Mean portion size (g) of meat were across all meat-eating occasions. Detailed methodology is provided in the Online Methods.
