# Supplementary material for: Smaller meat portions contribute the most to reducing meat consumption in the United Kingdom
Source: Nat Food. 2024 Nov 1;5(12):982–7. doi: 10.1038/s43016-024-01070-2 (PMC11655353; doi:10.1038/s43016-024-01070-2)
Supplement: Supplementary file 2 — Reporting Summary [file 43016_2024_1070_MOESM2_ESM.pdf]

nature portfolio

Corresponding author(s): Alexander Vonderschmidt

Last updated by author(s): Sep 1, 2024

Reporting Summary

Nature Portfolio wishes to improve the reproducibility of the work that we publish. This form provides structure for consistency and transparency in reporting. For further information on Nature Portfolio policies, see our Editorial Policies and the Editorial Policy Checklist.

Statistics

For all statistical analyses, confirm that the following items are present in the figure legend, table legend, main text, or Methods section.

n/a	Confirmed	
☐	☒	The exact sample size (n) for each experimental group/condition, given as a discrete number and unit of measurement
☐	☒	A statement on whether measurements were taken from distinct samples or whether the same sample was measured repeatedly
☐	☒	The statistical test(s) used AND whether they are one- or two-sided<br>*Only common tests should be described solely by name; describe more complex techniques in the Methods section.*
☐	☒	A description of all covariates tested
☐	☒	A description of any assumptions or corrections, such as tests of normality and adjustment for multiple comparisons
☐	☒	A full description of the statistical parameters including central tendency (e.g. means) or other basic estimates (e.g. regression coefficient) AND variation (e.g. standard deviation) or associated estimates of uncertainty (e.g. confidence intervals)
☐	☒	For null hypothesis testing, the test statistic (e.g. F, t, r) with confidence intervals, effect sizes, degrees of freedom and P value noted<br>*Give P values as exact values whenever suitable.*
☒	☐	For Bayesian analysis, information on the choice of priors and Markov chain Monte Carlo settings
☒	☐	For hierarchical and complex designs, identification of the appropriate level for tests and full reporting of outcomes
☒	☐	Estimates of effect sizes (e.g. Cohen's d, Pearson's r), indicating how they were calculated

Our web collection on statistics for biologists contains articles on many of the points above.

Software and code

Policy information about availability of computer code

Data collection	No software was used to collect data; secondary analysis of open-source data only.
Data analysis	All code used for analysis in this paper is open access on GitHub: https://github.com/axvonder/NDNSMeatTrends. All analyses were performed in R version v4.3.3, using the “survey" (v4.4.1) and “srvyr” (v1.2.0) packages to account for survey weighting in the demographic and regression analyses.

For manuscripts utilizing custom algorithms or software that are central to the research but not yet described in published literature, software must be made available to editors and reviewers. We strongly encourage code deposition in a community repository (e.g. GitHub). See the Nature Portfolio guidelines for submitting code & software for further information.

Data

Policy information about availability of data

All manuscripts must include a data availability statement. This statement should provide the following information, where applicable:

- Accession codes, unique identifiers, or web links for publicly available datasets
- A description of any restrictions on data availability
- For clinical datasets or third party data, please ensure that the statement adheres to our policy

This analysis used data from the National Diet and Nutrition Survey (NDNS) Rolling Programme years 1-11 (2008/09–2018/19). This data is open access and available for download from the UK Data Service: https://beta.ukdataservice.ac.uk/datacatalogue/series/series?id=2000033.

Research involving human participants, their data, or biological material

Policy information about studies with human participants or human data. See also policy information about sex, gender (identity/presentation), and sexual orientation and race, ethnicity and racism.

Reporting on sex and gender	From the NDNS methods of data collection: participants were asked to self-identify as either male or female, and in cases of non-disclosure, the interviewer reported sex.
Reporting on race, ethnicity, or other socially relevant groupings	We used household income as a covariate in our model and for population subgroup analysis. Per the NDNS, participants reported their total household income from the previous 12 months, before deductions and tax, inclusive of housing benefits and child allowance. Within the NDNS data files, these data were equivalised, accounting for household size and composition, and split into tertiles. Household income was included because previous studies of meat consumption in the NDNS (and in the literature more broadly) have shown differences in consumption patterns by household income (or other markers of income such as socioeconomic status).
Population characteristics	Sociodemographic variables included self-reported age, sex, and equivalised household income tertiles. For age, participants were asked to provide their date of birth, or age at last birthday if unknown; with interviewer estimates used if participants were unable or unwilling to provide this information. We categorised participants into the age groups of children (<18 years) and adults (≥18 years). Participants were asked to self-identify as either male or female, and in cases of non-disclosure, the interviewer reported sex.
Recruitment	Information on recruitment has been described in the NDNS methodology appendices: Bates B, Collins D, Cox L, et al. Appendix B methodology for year 9 of the NDNS RP. National diet and nutrition survey years 1 to 9 of the rolling programme (2008/2009 - 2016/2017): time trend and income analyses. Published online 2019.
Ethics oversight	Ethics considerations surrounding collection of National Diet and Nutrition Survey data are described in the National Diet and Nutrition Survey appendices (Appendix B; https://s3.eu-west-2.amazonaws.com/fsa-catalogue2/NDNS+Y1-9_Appendix +B_Methodology_FINAL.pdf).

Note that full information on the approval of the study protocol must also be provided in the manuscript.

Field-specific reporting

Please select the one below that is the best fit for your research. If you are not sure, read the appropriate sections before making your selection.

☐ Life sciences ☒ Behavioural & social sciences ☐ Ecological, evolutionary & environmental sciences

For a reference copy of the document with all sections, see nature.com/documents/nr-reporting-summary-flat.pdf

Behavioural & social sciences study design

All studies must disclose on these points even when the disclosure is negative.

Study description	Our study is a quantitative secondary analysis of an open-sourced, survey-weighted and nationally representative dataset (National Diet and Nutrition Survey, NDNS) in the UK.
Research sample	The research sample (NDNS) is a nationally representative and weighted cohort of UK individuals; aged 1.5 - 96. The dataset is existing and open-sourced. This dataset was chosen as it is the only dataset which has collected nationally representative diet data in the UK.
Sampling strategy	No formal sample size calculation was performed for this study. The sample size was determined based on the design and methodology of the NDNS, which aims to be nationally representative of the UK population. The NDNS employs a stratified random sampling strategy to ensure the inclusion of diverse demographic groups across the UK, with survey weights applied to account for population distributions and non-response. The sample was drawn from Postcode Address Files, which were grouped into Primary Sampling Units (PSUs) based on postcode sectors. From each PSU, a list of addresses was randomly selected, and the interviewer randomly selected up to 1 adult and 1 child to take part from each household. The use of a large, nationally representative sample of over 15,000 individuals across multiple years enhances the robustness and generalisability of the findings. The repeated measures over a decade also provide a comprehensive overview of dietary behaviours over time, which is particularly valuable for trend analysis. Therefore, the extensive coverage and methodological rigor of the NDNS provide confidence that the sample size is sufficient to detect meaningful trends and differences in meat consumption behaviours across the UK population.
Data collection	Specific to our analysis, dietary data were collected using 4-day, consecutive food diaries, with the survey design ensuring equal representation of all days of the week. Briefly, participants were instructed to record all food and beverages they consumed over the assigned 4-day period within a paper journal. Participants estimated portion sizes using household measures (e.g., tablespoons) or reporting the weights on food labels. Further information on data collection for the NDNS can be found in the NDNS appendices: https://assets.publishing.service.gov.uk/media/6149e831e90e070434bbc0da/Follow_up_study_2020_Appendix_A_Methodology.pdf<br><br>More broadly, the NDNS is public health surveillance tool designed to collect comprehensive dietary data without any specific hypothesis testing in mind at the time of data collection. Thus, this large-scale observational study that does not involve an experimental condition, and therefore, researcher blinding to experimental conditions was not applicable. Both the NDNS and this

	analysis did not involve an intervention, but rather focused on observing and analysing existing dietary behaviours in the UK population over time.
Timing	Data for each survey year was collected during fieldwork in multi-month waves throughout each respective round of the NDNS. For years 1-4, data was collected from April 2008 to March 2011. For years 5-6, data was collected from April 2012 to June 2014. For years 7 & 8, data was collected from April 2014 to June 2016. For years 9-11, data was collected from April 2016 to June 2019. Information on timing of data collection for the NDNS can be found in the NDNS appendices. Specifically, Appendix B for each respective NDNS round contains detailed information on data collection timing.
Data exclusions	As this study explored the frequency of days in which meat was consumed, participants with <4 food-diary days were not included in the analyses (n=323, 2%). This exclusion was established before analyses were completed.
Non-participation	In general, the NDNS ensured high participation rates and low drop out through many mechanisms such as: comprehensive communication (descriptive letters prior to survey), incentives, flexibility in participation and interviewing over longer time periods, follow-ups and reminders (through phone calls, emails, and letters), simplified data collection, training, confidentiality assurances, and building community networks/relationships. Exact participation varied by year of the Survey, though for year 1 (2008/09), participation was very high at 96% of selected individuals being "full productive respondents." Information on participation statistics during data collection/interviews for the NDNS can be found in the NDNS appendices: https://assets.publishing.service.gov.uk/media/6149e831e90e070434bbc0da/Follow_up_study_2020_Appendix_A_Methodology.pdf as well as comparison studies of the NDNS assessment method: https://www.mrc-epid.cam.ac.uk/wp-content/uploads/2021/07/NDNS-Comparison-Study-report_FINAL.pdf
Randomization	Households were randomly selected from each PSU based on postcode sector. Within a randomly selected household, 1 adult and 1 child was randomly selected. This study (as well as the NDNS itself) did not involve the creation of experimental groups, as it is based on observational data from the NDNS. The random selection of households and participants within households was conducted to ensure that the sample was representative of the UK population. Thus, random allocation and control of covariates in the context of group assignments are not applicable to this study. This analysis controlled for potential confounding factors through survey weighting during data analysis, ensuring that the results are representative and account for demographic differences across the population.

Reporting for specific materials, systems and methods

We require information from authors about some types of materials, experimental systems and methods used in many studies. Here, indicate whether each material, system or method listed is relevant to your study. If you are not sure if a list item applies to your research, read the appropriate section before selecting a response.

Materials & experimental systems

n/a	Involved in the study
☒	☐ Antibodies
☒	☐ Eukaryotic cell lines
☒	☐ Palaeontology and archaeology
☒	☐ Animals and other organisms
☒	☐ Clinical data
☒	☐ Dual use research of concern
☒	☐ Plants

Methods

n/a	Involved in the study
☒	☐ ChIP-seq
☒	☐ Flow cytometry
☒	☐ MRI-based neuroimaging

Plants

Seed stocks	*Report on the source of all seed stocks or other plant material used. If applicable, state the seed stock centre and catalogue number. If plant specimens were collected from the field, describe the collection location, date and sampling procedures.*
Novel plant genotypes	*Describe the methods by which all novel plant genotypes were produced. This includes those generated by transgenic approaches, gene editing, chemical/radiation-based mutagenesis and hybridization. For transgenic lines, describe the transformation method, the number of independent lines analyzed and the generation upon which experiments were performed. For gene-edited lines, describe the editor used, the endogenous sequence targeted for editing, the targeting guide RNA sequence (if applicable) and how the editor was applied.*
Authentication	*Describe any authentication procedures for each seed stock used or novel genotype generated. Describe any experiments used to assess the effect of a mutation and, where applicable, how potential secondary effects (e.g. second site T-DNA insertions, mosiacism, off-target gene editing) were examined.*
